# The effect and biomechanical mechanisms of intradermal needle for post-stroke hemiplegia recovery: Study protocol for a randomized controlled pilot trial

**DOI:** 10.1097/MD.0000000000010448

**Published:** 2018-04-20

**Authors:** Ruyu Yan, Yong Zhang, Jingyang Lim, Fan Yang, Li Zhou, Diyang Lyu, Yahui Wang, Yihuai Zou, Zongheng Li

**Affiliations:** a Department of Rehabilitation; b Department of Neurology and Stroke Center, Dongzhimen Hospital affiliated to Beijing University of Chinese Medicine; c Beijing University of Chinese Medicine, International College, Beijing, China.

**Keywords:** Biomechanical mechanisms, Hemiplegia, Intradermal needle, Stroke rehabilitation

## Abstract

The incidence of stroke has increased significantly in recent years. Post-stroke hemiplegia is a common stroke complication with long-term negative consequences. Several studies have suggested that acupuncture could be an effective intervention for the rehabilitation of post-stroke hemiplegia. Intradermal needling is a kind of acupuncture which is widely used in clinical settings. This study attempts to investigate the biomechanical effects of intradermal needle for post-stroke hemiplegia recovery.

This proposed study is a single-centered, prospective, single-blinded (patient-assessor-blinded), randomized clinical pilot trial involving 40 patients with post-stroke hemiplegia. Patients will be randomized to an experimental group or control group in a 1:1 ratio. All of them will receive conventional rehabilitation therapies. Patients in the experimental group will be treated with intradermal needle, whereas patients in the control group will receive sham intradermal needle. The primary outcome measures will be biomechanically validated from the parameters of RSSCAN gait system: plantar pressure distribution, step length, and stride. The scores of clinical scales such as National Institutes of Health Stroke Scale, Fugl-Meyer Assessment, Berg Balance Scale, Barthel Index, and Stroke-specific Quality of Life Scale will be assessed as secondary outcome measures. All assessments will be conducted at baseline, 4 weeks after intervention and at the end of 3 months’ follow-up.

The purpose of this study is to explore the potential effect and biomechanical mechanisms of intradermal needle for post-stroke hemiplegia recovery, as well as to provide a basis for future larger clinical studies.

## 1 Background

Stroke has become a leading cause of disability and the second most common cause of death, which amounts to a huge combined burden worldwide.^[1,2]^ In China, there are more than seven million stroke survivors. Approximately, 70% of them are experiencing functional disabilities. Hemiplegia is the most significant symptom.^[3]^ People suffering from hemiplegia face a variety of challenges in regaining independence in daily activities, which not only requires long-term recovery but also increases the burden on the family and society as a whole. It is therefore of great benefit to seek an effective rehabilitation treatment for post-stroke hemiplegia.

Other than the comprehensive rehabilitation techniques, acupuncture is used widely in clinical settings and recommended by the World Health Organization as an alternative and complementary therapy.^[4]^ Many clinical studies about acupuncture for post-stroke hemiplegia have been conducted in the past decades. There is growing evidence confirming that acupuncture can contribute to more patients recovering from akinetic apraxia, regaining muscle strength, improving motor function and returning society.^[5,6]^ Different methods of acupuncture have varying influences. Current acupuncture therapy in common use includes body acupuncture, scalp acupuncture,^[7]^ electro-acupuncture,^[8]^ among others. These kinds of treatment can only stimulate an acupoint temporarily, instead of the full length of time required for the patients’ recovery. Under this context, it is necessary to figure out other methods to extend the time of acupuncture stimulation and enhance the therapeutic effect.

Intradermal needle, a kind of acupuncture, meets the above requirement. This therapy has been developed to treat various diseases because of its distinct advantages in recent years.^[9–12]^ It can be inserted perpendicularly into the skin with a tiny needle and be fixed with a piece of adhesive tape for 1 to 5 days, during which time the patients can move freely without any discomfort. It can provide continuous stimulation to the area, so as to promote effectiveness. To the best of our knowledge, no randomized controlled trials have been performed to demonstrate the clinical curative effect of intradermal needle on post-stroke hemiplegia. In the present study, we will focus on this point and further elucidate it.

In previous work, patients’ motor function and life ability were assessed by scales, which lacks objectivity because the score depends on the researchers partly. Very few studies focused on the objective measure of evaluation.^[13–15]^ The clinical intervention strategies which are used must be backed by a high level of assessment-based support. The RSSCAN gait system can provide biomechanical parameters such as dynamic plantar pressure distribution, step length, foot impulse curve, walking support phase, pace, stride frequency, among others.^[16]^ Because it is a reliable means for foot-examination quantitatively and automatically, it had been used to detect the gait parameters for distinguishing between normal and pathological gait,^[17]^ classifying foot types,^[18]^ even assessing the effect of foot surgery.^[19]^ We mainly use the RSSCAN gait system to evaluate the motor function of lower limb in the present work.

Based on the above, we conceived this clinical pilot trial with the following primary aims. First is to collect preliminary data to observe whether intradermal needle add-on therapy leads to a more effective recovery for patients with post-stroke hemiplegia. Second is to adopt objective outcome measures to elucidate the biomechanical mechanisms underlying the effects.

## 2 Methods/design

### 2.1 Study design

**Figure 1:**
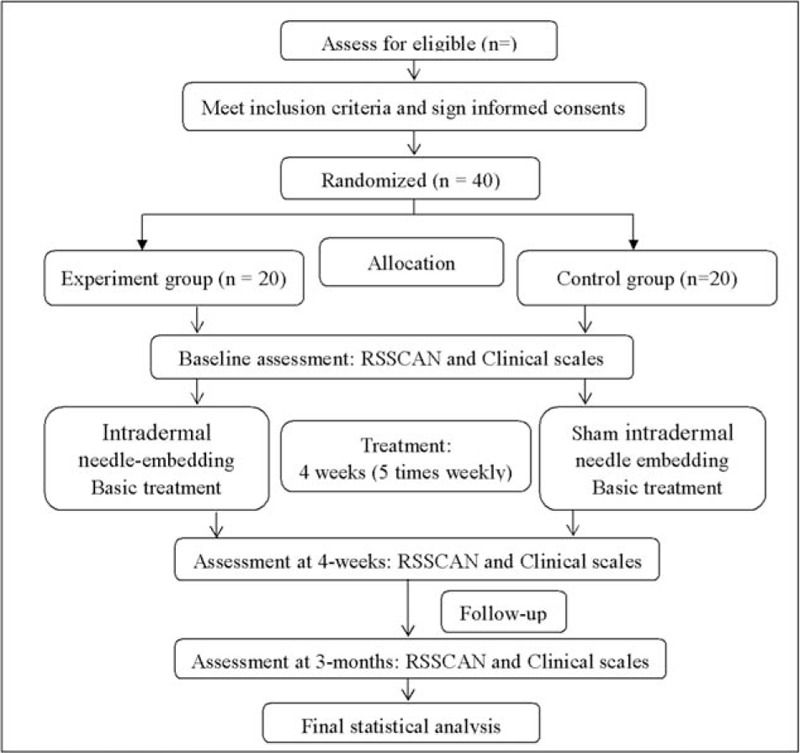
Flowchart of the study design.

**Figure 2:**
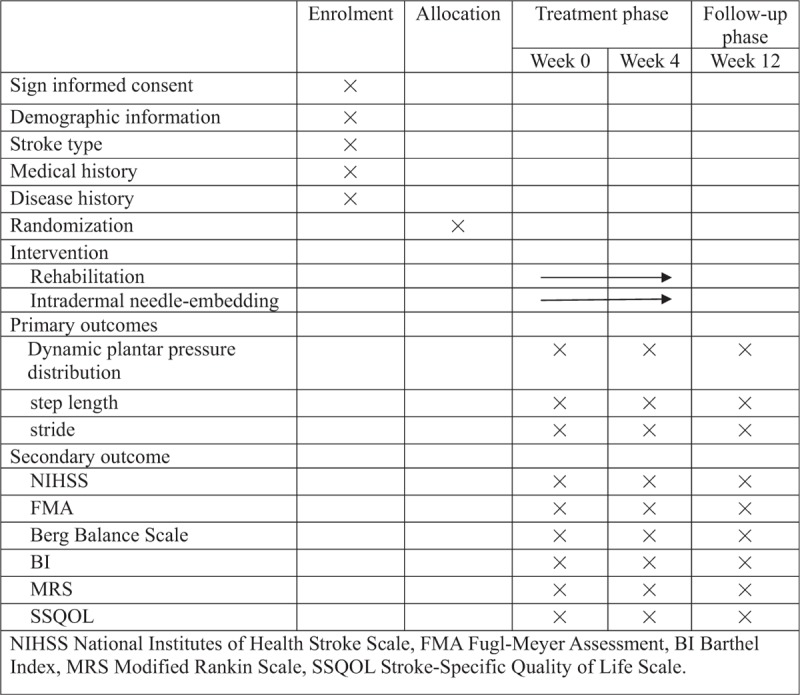
Timing of treatment visits and data collection.

This study is a prospective, single-centered, randomized, patient-assessor-blinded, parallel-controlled clinical trial. The chief investigator and Clinical Trials Group (CTG) will take responsibility for the overall management of this trial, including the study design, coordination, monitoring, data analysis and results reporting. Any changes to the study protocol will be submitted to them. They will decide whether it is necessary to change or even stop the protocol. We began this study after having discussed in its entirety with each member of the research team. The patients according to inclusive criteria of Dongzhimen Hospital will be recruited and then invited to participate in this study. The eligible participants will be randomly allocated into two groups by a 1:1 ratio using a randomization procedure. All patients will receive conventional rehabilitation therapies. On top of basic treatment, the experimental group will receive authentic intradermal needling, whereas the control group will receive sham intradermal needling. All therapeutic methods will be taken once a day, 5 days per week. This study is designed to coincide with routine care as much as possible and align with follow-up visits. After 4 weeks of treatment and 3-month follow-up, we will evaluate the effects and prognosis by biomechanical indexes from RSSCAN gait system and clinical scales. Our trial design is summarized in Figure 1. The study timeline and event schedule according to the Standard Protocol Items: Recommendations for Interventional Trials (SPIRIT) 2013 Statement^[20]^ are detailed in Figure 2.

### 2.2 Ethical issues

Our protocol is in accordance with principles of the Declaration of Helsinki^[21]^ and has been approved by the Research Ethical Committee (REC) of Dongzhimen Hospital, the first affiliated teaching hospital of Beijing University of Chinese Medicine (reference number: ECPJ-BDY-2016–76 ). The protocol has been registered with the Chinese Clinical Trial Registry: ChiCTR-IPR-17011989.

### 2.3 Sample size

Out study is a preliminary clinical trial aiming to evaluate the feasibility of intradermal needle therapy for post-stroke hemiplegia recovery. Therefore, we did not determine sample size basing on statistical calculations.^[22]^ An alternative approach to estimate the sample sizes for pilot studies with 10 to 40 patients per group was adopted.^[23]^ Accordingly, to exceed the minimal number and combine with the actual situation, a total of 40 patients will be recruited in this research, 20 in each group. Assuming a 20% drop-out rate, we aimed to recruit 50 patients. Each group will require 25 initial participants.

### 2.4 Participant recruitment

Participants will be recruited from Dongzhimen Hospital affiliated with the Beijing University of Traditional Chinese Medicine in Beijing, China. Our study will be promoted via leaflets and through the Internet. We will communicate with prospective participants about study details. If they are interested in participating and willing to sign the informed consent, they will be invited into this study.

### 2.5 Informed consent

We will fully explain the details of this study to participants and their families before they take part in this research, including trial objectives, characteristics, probable benefits, potential risks, as well as the obligations as stated in the Declaration of Helsinki *2013*.^[21]^ They will also be informed that their participation in the trial is entirely voluntary and that they can withdraw at any time for any reason. All patients who meet all inclusion criteria and none of exclusion criteria and agree with this trial will give their written informed consents before they undergo any interventions related to this study. Their personal information will be always undisclosed and kept confidential.

### 2.6 Inclusion criteria

Participants satisfying the following inclusion criteria will be included: confirmed stroke patients with results from computed tomography or magnetic resonance imaging; aged 35 to 75 years; first-ever stroke or recurrent stroke without neurofunctional disablility (score of modified Rankin Scale ≤2); stable condition after stroke and within 3 months of the duration; with hemiplegia and be able to walk at least 6 m; sufficient cognition to follow commands, Mini-Mental State Examination (MMSE) score >24; never used intradermal needle before; the voluntary informed consent form signed.

### 2.7 Exclusion criteria

Participants with any of the following exclusion criteria will be excluded: received thrombolytic therapy or surgery; stroke during 3 months after the onset; stroke without hemiplegia or with but could not walk 6 m; vital signs are not stable or with worsening conditions; with other diseases such as osteoarthrosis that will affect motor function of lower limbs; used intradermal needle before; pregnant or lactating women; participating in other clinical trials.

### 2.8 Randomization and allocation concealment

All participants will be assigned to experimental group or control group in a 1:1 ratio according to a computer-generated randomization list by an independent researcher. In accordance with best practice recommendations for randomized controlled trials, allocation concealment will be employed. Assignments will be sealed in opaque envelopes by a nurse who will be trained before the trial and will not participate in treatment. As only the therapist who opens the envelopes will assign patients to a different group. It is impossible to predict and interference the assignments factitiously. The participants will be blinded. They will be told that they have been randomly allocated to one group, and be treated with regular rehabilitation therapies. Moreover, the outcome assessors and data statistical analysts will remain blinded to the intervention methods. Unblinding will be considered when adverse events (AEs) occur.

### 2.9 Interventions and comparison

#### 2.9.1 Experimental group

**Figure 3:**
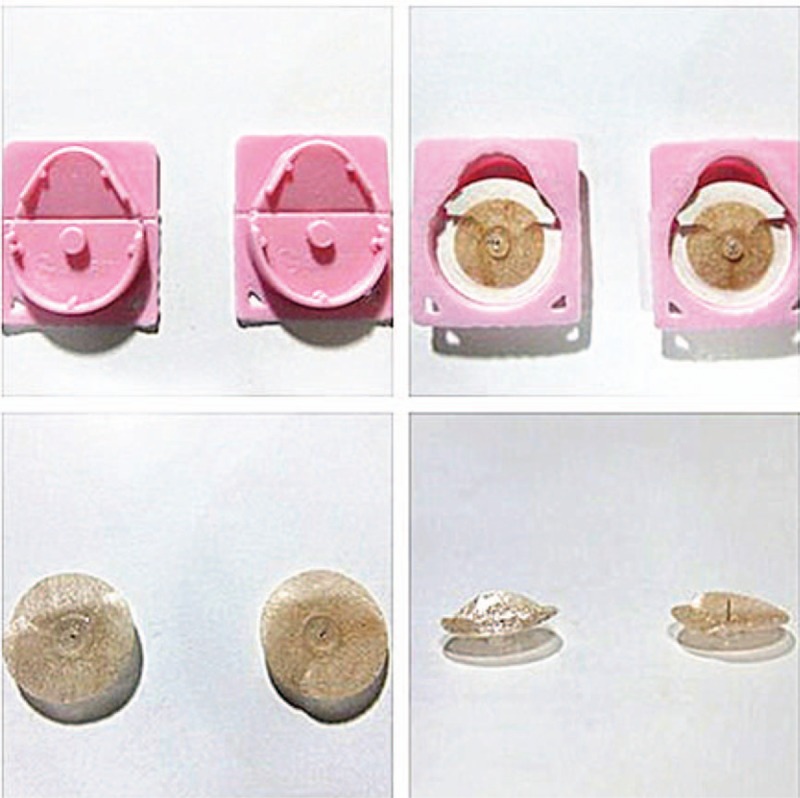
Intradermal needles (sham: left, authentic: right).

Patients allocated to the group will receive conventional rehabilitation programs as control group. As the aim of our study is to analyze the effects of intradermal needle for stroke hemiplegia recovery, participants in this group will be treated by intradermal needle-embedding in addition. The appearance of intradermal needle is in Figure 3. According to a summary of previous investigation^[24–26]^ and clinical experience, taking into account the patients’ normal activities while avoiding contact with shoes and socks, we selected following acupoints from the affected side for needling: Liangqiu (ST34), Yanglingquan (GB34), Yinlingquan (SP9), Xuanzhong (GB39), Fuyang(BL59), Sanyinjiao (SP6). The intradermal needle will complies with the Standardize Manipulations Of Acupuncture And Moxibustion-Part 8: Intradermal Needle (GB/T 21709.8–2008).^[27]^ All acupoints are located according to the World Health Organization standard acupuncture point locations.^[28]^

The intradermal needles (0.2 × 1.5 mm, Seirin, Japan) (Fig. 2) are disposable and sterile, with waterproof adhesive tape above which can help to affix the needle beneath. Patients after modern rehabilitation training will be inserted with these needles in a perpendicular fashion. In comparison to typical traditional acupuncture, intradermal needles are smaller, less deeply, and with less manipulation. All needles will be replaced after treatment the next day. In other words, patients will carry these intradermal needles for the whole course of therapy. The intradermal needle treatment will be implemented once a day from Monday to Friday, lasting 4 weeks. To ensure the optimal effects of needle stimulation, our therapists will be required to have clinical experience of acupuncture treatments for at least 8 years. The items are presented in the Standards for Reporting Interventions in Clinical Trials of Acupuncture (STRICTA).^[29]^

#### 2.9.2 Control group

Patients allocated to the control group will receive conventional rehabilitation programs for post-stroke hemiplegia. Therapies are based on neurodevelopmental theory and will include position of limb, passive and active assisted activities, postural balance training, trunk controlling, task oriented practicing, among others.^[30]^ It will last approximately 1 hour every time. After this treatment, sham intradermal needles, which look similar at the surface but without needle underneath will be used. It can be adhered to the skin instead of being needle-inserted. The acupoints location and manipulations are taken equally with experimental group. The retention time and frequency are identical to authentic intradermal needle therapy. To ensure the homogeneity of research, all rehabilitation programs will be done by qualified therapists and we have trained them with rehabilitation technology before the implementation of the project (Fig. 1).

### 2.10 Follow up

After finishing the treatment of four weeks, follow-up will be done on all patients for three months. Because of the specificity of stroke patients’ recovery, they may accept other rehabilitation treatments during this period. Considering the ethical issue, we will not intervene in this except for the prohibition of additional intradermal needle. We have created rehabilitation diaries and telephone interview forms (TIFs). We will train and ask each patient to record their condition every day after hospital discharge, such as medication used, method and frequency of treatment, AEs, and so on. At the end of each month, our assessors will call patients to enquire their conditions based on the TIFs. At the end of follow-up, participants will be referred for RSSCAN gait system and clinical scales as before. All forms will be collected by the data collectors regardless whether they have withdrawn from the trial assessments for reviewing at the end of this trial.

### 2.11 Outcome measures

The participations will be examined at baseline, reexamined after 4-week treatment, and again at the end of 3 months’ follow-up. Data will be assessed by trained, certified assessors.

### 2.12 Basic characteristic variables

We will conduct demographic information of all the participants at baseline assessments, including sex, age, time from the onset of stroke, medical history, clinical syndrome (left or right hemiplegia), diseased location, and other detailed information. Vital signs such as the resting blood pressure, pulse, respiration rate, and body temperature will be measured every day by nurses.

### 2.13 Primary outcome measurement

In the prospective trial, the primary outcome will be the ability of walking, measured by the data from RSSCAN gait system (2 × 0.4m, Olen, made in Belgium). It is a dynamic plantar pressure system, including a walking plate with more than 16,000 sensors distributed, a Footscan 3D box and other supporting software. It is sensitive in detecting parameters when people stand or walk on the sensor array plate. In our study, we will collect static and dynamic foot-parameters of dynamic plantar pressure distribution, step length, and stride as primary outcome. Plantar pressure distribution includes the peek pressure (PP) in 10 regions of foot: hallux, toes 2 to 5, first to fifth metatarsals, midfoot, medial heel, and lateral heel. PP is the most relied and common used upon plantar pressure parameter.^[31]^ We will observe PP of these 10 regions and the changes after treatment. Step length and stride can reflect the self-selected walking distance.^[32]^ A cross-sectional study indicated that step length symmetry demonstrated a systematic linear trend toward greater asymmetry in groups in the later stages of post-stroke.^[33]^ So we will measure these indexes to evaluate intervention as well.

According to the manufacturer's manual, the RSSCAN gait system will be calibrated before each individual's test. Patients’ weight and foot size will be recorded into computer for calibration. Patients will be instructed to get familiar with the walking plate every time before the testing. They will be told to walk at their comfortable pace and look straight ahead. A walking phase to be considered as reliable when meets the following criteria: at least 1 complete footprint for each foot, a heel-strike pattern, no obvious adjustment in gait pattern to contact the plate. We will select the most representative step of each foot to analysis. All tests will be done by the same RSSCAN system operator, who has received standardized training before.

### 2.14 Secondary outcome measurement

The scores change of clinical scales will be the secondary outcome measurement. First is NIHSS (National Institutes of Health Stroke Scale), which is a standardized scale to describe neurological deficits in stroke patients. Fugl-Meyer Assessment is usually used to evaluate motor function. It includes an assessment of the upper extremity and lower extremity. This study tends to emphasize the patients’ gait, so we selected the part of the lower limbs only, with a total score of 34. Most patients with post-stroke hemiplegia have trouble in balance control; Berg Balance Scale was developed as a tool for quantifying the ability of keeping balance. Barthel Index, Modified Rankin Scale, and Stroke-specific Quality of Life Scale indicate the capacity of daily living.

### 2.15 Incidence of AEs

Any related and unexpected AEs happened during this study will be recorded in detail, and the occurrence of these will be analyzed. Serious AEs will be reported to the chief investigator, CTG, and REC immediately. They will make decision on whether the participant needs to withdraw from the trial or the study should be adjusted. Those who suffer accidental injury from our trial will be provided free treatment until they recover.

### 2.16 Data management and monitoring

Before the study, we have set up Data Management and Monitoring Committee (DMC) and trained all researchers about data management. DMC is independent from trial investigators, and has no competing interests. First, all assessors will be responsible for the assessment and acquisition of participants’ information during the study. After they finish the Case Report Forms and TIFs completely, 2 data collectors will convert these and rehabilitation dairies to electronic data. All these original data related to the research including paper forms and electronic documents will be archived securely in the Clinical Research Center of Dongzhimen Hospital. Only researchers in our study team will have access to the final complete data, others who have any questions will be required written requests to our data manager to be permitted.

Monitoring is another role and responsibility of DMC. Members of DMC will monitor the overall quality and completeness of the data, examine original documents, interview assessors, and confirm that the study is complied with the principles of this protocol. The monitors will verify that all AEs be recorded in the correct format.

### 2.17 Auditing

Research Department of Dongzhimen Hospital will in charge of auditing, which is independent form trial investigators. The processes reviewed include participant enrolment, consent, eligibility, allocation to study groups, adherence to trial interventions, policies to protect participants, completeness, accuracy, and timeliness of data collection. The periodic review is every 2 months.

### 2.18 Statistical analysis

Statisticians will be responsible for the statistical analysis. The main analysis will compare the changes in all outcome measurements of between-group or within-group to evaluate the effect of intradermal needle treatment. An intention-to treat analysis will be conducted using the SPSS (Statistical Product and Service Solutions) program (version 22.0, Chicago, IL). Continuous variables will be expressed as means ± standard deviations, Wilcoxon rank sum tests with no adjustment for multiple comparisons will be used for comparisons. Categorical variables will be presented with frequencies or percentages. Fisher exact test will be applied in testing blinding between groups because of small cell counts. A *P* value of <.05 (2-sided) is considered to indicate statistical significance, with 95% confidence intervals.

### 2.19 Dissemination

The result of this study will be published regardless of the direction of the effect.

## 3 Discussion

Hemiplegia is the most common symptom of stroke. The typical feature of this disease is that the apraxia will exist and continue for several weeks, months, or years. It causes a heavy burden on both healthcare resources and the society. Cohort studies have consistently reinforced the importance of post-stroke rehabilitation to stimulate recovery.^[34]^ Acupuncture is considered as a frequently used therapy to harmonize and regulate the functional activities of the brain and body. Previous studies have indicated that acupuncture combined with conventional rehabilitation therapies is more effective for post-stroke patients.^[35]^ However, the treatment of acupuncture is carried out at clinic, lasting temporarily. Patients cannot receive any therapeutic measures besides this period. Thus, therapies that can maintain for longer time are required.

Intradermal needle, with the advantages of convenience, little pain, long-lasting, satisfactory effects, and few adverse reactions, is attracting increasing clinical attention.^[36]^ It is inserted and affixed in superficial skin, which is densely packed with sensory and motor neurons. The nerves of skin are associated with brain. It can be transmitted to central nervous system when someone stimulates the acupoints on the skin. The cerebral center integrates the information and corresponds through homologous movement.^[37]^ In the aspect of different needle-retaining, some researchers randomly divided 251 ischemic apoplexy patients into 20, 40, and 60-minute groups. Through the results of hemorheological values, the therapeutic effect of 60-minute group was the best.^[38]^ Intradermal needle is a kind of superficial needling, which can be maintained for 1–3 days, so as to extend the effect of acupuncture. It is therefore of great clinical significance that our study uses intradermal needle therapy to treat post-stroke hemiplegia.

In clinical studies, evaluations play an important role. A majority of previous studies adopt clinical rating scales to estimate the therapeutic effect, which have been proven to be prone to some errors associated to the evaluators’ subjective perception.^[39]^ Lack of objective assessments affects the quality of results.^[40]^ To overcome this limitation, this study may potentially confirm whether or not intradermal needle is effective for post-stroke hemiplegia by using biomechanical outcome measurements from the RSSCAN gait system. It can provide objective kinematic data of static and dynamic gait. Vertical plantar pressure dispersion of the foot will be recorded, processed, and graphically displayed in terms of sequential gait changes.^[41]^ It helps to remove some of the unavoidable guess work from essential diagnostic and therapeutic procedures. Our previous work has shown that it is affordable, accurate, and reliable for the assessment of motor function in patients with stroke.^[42]^

The unavoidable limitations of our trial consist of three domains. First, despite the assessor-blinding, some patients who got treated with intradermal needles will likely know which group they are in. Therefore, we will select participants who have never used these needles and keep participants separate from each other during the treatment procedure. Next, after completion of the 4-week treatment, a 3-month follow-up will be conducted. This time span means that participants’ compliance could be a problem. Thus, we designed a recover diary and regular telephone follow-up to minimize this situation. The last limitation is the small sample size, as this trial was proposed to be a pilot study for further larger scaled clinical study.

This study focuses on the biomechanical effect of intradermal needle embedding for post-stroke hemiplegia, so far there is no study of this aspect. If the trial is successfully carried out, we may provide feasibility data and basic information for utilizing this therapy and build a stronger foundation for other studies.

### 3.1 Trial status

This trial started on 1 May 2016 and is currently in the participants’ enrollment.

## Acknowledgments

The authors thank all patients and doctors who participated in this trial for their cooperation. The authors also would like to express their gratitude to the anonymous reviewers for their excellent work and constructive criticisms.

## Author contributions

All authors participated in the conception and design of the initial protocol; R.Y. drafted the manuscript; Z.L. and Y.Z. revised the manuscript; Y.W. is in charge of patient recruitment; Y.Z. is the intradermal needle instructor; L.Z. will carry out the rehabilitation programs; R.Y. is responsible for assessing clinical scales; F.Y. is the operator of the footscan gait system; D.L. is the statistician; all authors read and approved the final manuscript; Y.Z. contributed in the conceptualization; Y.W. contributed in data curation; R.Y. contributed in formal analysis; F.Y., L.Z. and D.L. contributed in investigation; Y.Z. contributed in project administration; R.Y. and Z.L. contributed in writing of the original draft; J.L. contributed in writing-review and editing.

**Conceptualization:** Yong Zhang.

**Data curation:** Yahui Wang.

**Formal analysis:** Ruyu Yan.

**Investigation:** Fan Yang, Li Zhou, Diyang Lyu.

**Project administration:** Yihuai Zou.

**Writing – original draft:** Ruyu Yan, Zongheng Li.

**Writing – review & editing:** Jingyang Lim.
